# Estimation of regional and at-site quantiles of extreme winds under flood index procedure

**DOI:** 10.1016/j.heliyon.2023.e23388

**Published:** 2023-12-07

**Authors:** Ishfaq Ahmad, Touqeer Ahmad, Usman Shahzad, Muhammad Athar Ameer, Walid Emam, Yusra Tashkandy, Zanib Badar

**Affiliations:** a Department of Mathematics and Statistics, Faculty of Basic and Applied Sciences, International Islamic University, 44000, Islamabad, Pakistan; b CREST-ENSAI, 51 Rue Blaise Pascal, 35170, Bruz, France; c Department of Statistics and Operations Research, Faculty of Science, King Saud University, P.O. Box 2455, Riyadh, 11451, Saudi Arabia; d Government Associate College for Girls, Chak-Beli, Rawalpindi, Pakistan

**Keywords:** Linear moments, Monte Carlo simulation, Quantile estimates, Wind speed, Regional frequency analysis

## Abstract

Extreme winds are becoming more common among environmental events with the most catastrophic societal consequences. A regional frequency analysis of Daily Annual Maximum Wind Speed (DAMWS) is necessary not only for a comprehensive understanding of wind hazards but also for infrastructure design and safety, wind energy potential, disaster risk reduction, insurance and risk assessment in a particular region of study. This study investigated regional frequency analysis of DAMWS of Baluchistan and Sindh provinces of Pakistan. L-moments regionalization techniques along with flood index procedure were applied to DAMWS records of 21 stations from 1990 to 2019 across the study area. We intended to find the regional frequency distribution for maximum winds and predict the returns for extreme winds events in the future. Only one station namely Lasbella was found to be discordant. With the help of cluster analysis, the remaining 20 stations were further divided into two homogeneous. Heterogeneity measures validate that both regions are homogenous with allotted stations. Regional quantiles for both regions are estimated through best-fit probability distribution among Generalized Normal (GNO), Generalized Logistic (GLO), Pearson Type 3 (P3), Generalized Pareto (GPA), and Generalized Extreme Value (GEV). Robustness of GLO distribution compared to GEV distribution is assessed through Monte Carlo simulations of relative bias and relative root mean square error. Findings clearly show that GLO distribution is the best for regional modeling. Furthermore, with the help of index flood procedure we determined at-site quantiles of all stations for various return periods. These estimated quantiles are of valuable information for various sectors, including infrastructure, energy, disaster management, and climate resilience, leading to improved planning, development, and risk reduction in the face of wind-related hazards in Sindh and Balochistan provinces of Pakistan.

## 1 Introduction

In dry and semi-arid regions of the world, wind is considered a prominent environmental phenomenon. In recent years, wind storms have become a major agricultural, ecological, and urban population issue. In the monsoon zone, Pakistan is greatly affected by wind storms, especially the areas in Sindh and Balochistan provinces. As an emerging state, Pakistan faces many devastations due to extreme wind speed, which causes financial loss, physical demolitions, and human and animal injuries. Therefore, wind speed modelling is essential for the country to overcome destructions linked with extreme wind events.

Accurate codification of extreme wind speed events is required in various design projects such as buildings, bridges, wind turbines and tower installation. True predictions of extreme winds are helpful to ensure the safety and reliability of the structures, and to save agricultural farms and human beings. On the other hand, wind speed is one of the most critical parameters to consider while building and running a wind turbine system [1].

In most cases, the frequency analysis of extreme winds through quantiles is intended by fitting statistical distributions to a sample of observed wind speed data. Frequency analysis of winds offers valuable information for individuals working in the field of structural and environmental design and renewable energy studies [2]. In the perspective of wind speed modelling, the Weibull distribution has been recommended widely in literature [[3], [4], [5], [6]]. In addition, the Gumbel and generalized extreme value (GEV) distributions are also commonly used for the analysis of extreme winds [[7], [8], [9], [10]]. There is no common consent for the choice of the modeling distribution, the performance may differ in different areas. Consequently, numerous extreme value models are incessantly debated and enhanced, for example, the multivariate compound extreme value distribution [11] peak-over-threshold [12]. When determining an appropriate equilibrium, precise calculation of the anticipated maximum wind speeds is critical. These estimations are often in the form of the quantiles value $x_{T}$, the extreme wind events that surpass once on average after every *T-year* return period [13].

The key drawback of at-site frequency analysis is that it suffers from sampling variability, which is especially problematic when estimating quantiles over long return periods [14,15]. In the case of small samples, at-site frequency analysis is not a reliable technique. Alternatively, we can obtain credible estimates of extreme events for future in the form of quantile estimates for policy implications by taking into account the Regional Frequency Analysis (RFA) of DAMWS [16]. The regional quantiles estimates are not of direct use for policy implications, so we implemented a flood index procedure to convert regional quantiles into at-site quantiles without performing at-site wind speed frequency analysis (ASWSFA) for individual sites.

Dalrymple (1960) [17] introduced the term index flood in the context of regional frequency study of flood flows. It refers to a scaling factor that is used to make data collected at different sites within a homogeneous region dimensionless so that it can be evaluated as a sample of regional data. Despite the connection to floods, the phrase index-flood is commonly used in regional frequency analysis of any sort of data. In this study, we considered the flood index method proposed by Ref. [17]. Despite of having some limitations due to statistical assumptions and the availability of historical data (sometimes not available), the index flood method has a number of advantages by giving a straightforward and very simple method for determining extreme wind speeds at specific locations. This is especially beneficial when there is a scarcity of data on extreme wind events at a certain place. As compared to more difficult and data-intensive procedures such as numerical modeling or reanalysis data, the flood index method can save significant time and money [18].

The objective of this study is to implement linear-moment-based RFA on DAMWS data of Sindh and Balochistan provinces of Pakistan. RFA provides us regional quantiles based on the best-fit probability distribution of a particular region but these quantiles cannot be used directly for policy implications at a particular site. So, instead of applying ASWSFA on extreme wind separately for various sites, we used flood index method to get more reliable at-site quantiles. These at-site quantiles would be of valuable information and could be used for various sectors, including infrastructure, energy, disaster management, and climate resilience, leading to improved planning, development, and risk reduction in the face of wind-related hazards in Sindh and Balochistan provinces of Pakistan. The study's novelty emphasizes the fact that no previous research has been undertaken in these areas, namely Sindh and Baluchistan, employing RFA along with index flood techniques. More specifically, the importance of these wind stations for this study stems from their proximity to coastal lines, which are particularly sensitive to extreme wind events such as Tropical Cyclones, Dust Storms, Squalls, and Coastal Windstorms. Understanding the frequency of these extreme wind events is critical for assessing the risk of wind-related hazards and further to quantify the chance of occurrence, which supports in the development of appropriate mitigation methods and emergency response plans.

Several international studies have been conducted in this direction over the last two decades, see, for instance, Refs. [[19], [20], [21], [22], [23]]. Different procedures had also been practiced by Refs. [[24], [25], [26], [27], [28], [29], [30], [31]]. Further, some studies related to wind modeling using Weibull distribution and Rayleigh distribution along with different estimation methods have been done in neighbouring country India [32,33]. The previous related work in Pakistan includes [[34], [35], [36], [37], [38]]. More relevantly, Fawad et al. [13] used seven distributions that analyze annual maximum wind speed and used different standards to determine the most suitable distribution for nine stations in Punjab province, Pakistan. In addition [[39], [40], [41]], used the ASFA procedure with a Bayesian framework for modelling extreme rainfall over the country. No study has been conducted before in the provinces, namely Sindh and Baluchistan, using ASWSFA and RFA approaches. More formally, the importance of these regions for this study is due to their linkage with coastal lines.

The paper is organized as follows. Section 2 deals with the materials and methods. For instance, details regarding the study area and observed data with their exploratory analysis are given in this section. Further, the procedure of regionalization is also presented in the same section. In Section 3, the results are discussed briefly. Conclusions are given in the final Section 4.

## 2 Material and methods

### 2.1 Study areas information

**Fig. 1:**
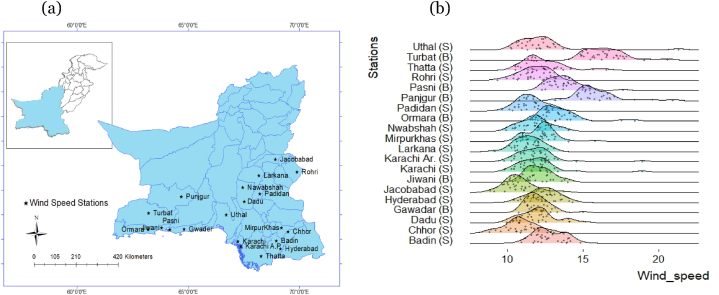
(a) Geographical existence of the stations used in this study. (b) Ridgeline plots of daily annual maximum wind data for all stations with climatic regions Sindh and Baluchistan.

Initially, this study used the daily annual maximum wind series (DAMWS) of twenty-one sites in Baluchistan and the Sindh provinces of Pakistan. Freely accessible DAMWS for all stations at a height of 10 m/s were downloaded from the NASA website. Daily maximum wind speed data consists of measurements from 00 h to 24 h throughout the day. Fig. 1 (a) shows the geographical locations of observed sites. We are interested in forming homogenous regions by using stations' characteristics (e.g., geographical closeness).

The descriptive statistics corresponding to all stations are explained in Table 1. Fig. 1 (b) shows the distribution (so-called ridgeline) plots of the DAMWS for observed stations with the respective provinces in parentheses. It can be observed from Fig. 1 (b) that the wind speed sometimes at some locations crosses 20 m/s. The maximum wind speed at Turbat and Punjgur stations are recorded more than 15 m/s many times. Also, the results of Table 1 indicate that the wind speed on average is higher at Turbat and Punjgur stations. These may happen due to the existence of these stations near the coastal line area of Baluchistan province.

**Table 1:** Information of observed stations and their exploratory analysis.

Station	Name	Latitude	Longitude	Elevation	Mean	SD	CV	Skewness	Kurtosis
1	Chhor	29.53	69.43	1046.90	10.77	1.10	0.06	0.02	0.16
2	Hydrabad	25.23	68.25	53.86	12.39	0.97	0.05	−0.02	0.12
3	Jiwani	25.04	61.48	30.69	12.11	0.85	0.08	0.11	0.09
4	Jacobabad	28.18	68.28	69.51	10.76	1.02	0.05	0.15	0.23
5	Karachi.A.P	24.54	67.08	48.58	12.18	1.16	0.05	0.17	0.22
6	Nawabshah	26.15	68.22	59.83	11.83	1.00	0.05	0.04	0.15
7	Punjgur	26.58	64.04	1037.86	15.87	1.60	0.05	0.24	0.28
8	Pasni	25.16	63.29	54.34	13.78	1.15	0.05	0.19	0.19
9	Badin	24.38	68.54	5.32	12.79	1.00	0.05	0.06	0.04
10	Gwadar	25.08	62.20	49.08	12.03	0.85	0.04	0.24	0.19
11	Larkana	27.32	68.14	51.26	11.37	0.89	0.05	−0.01	0.12
12	Padidan	26.51	68.08	45.41	11.54	0.92	0.05	0.07	0.14
13	Rohri	27.40	68.54	59.87	11.75	0.98	0.05	−0.12	0.09
14	Ormara	25.12	64.40	94.54	13.52	1.46	0.05	0.33	0.25
15	Turbat	25.59	63.04	254.44	16.48	1.31	0.04	0.17	0.14
16	Karachi	24.54	66.56	24.28	12.04	1.65	0.07	0.25	0.32
17	Dadu	26.43	67.47	357.64	12.07	1.24	0.06	0.10	0.18
18	Mirpurkhas	25.31	69.00	17.20	12.29	1.04	0.05	0.08	0.23
19	Thatta	24.45	67.54	5.97	12.37	1.40	0.06	0.24	0.20
20	Uthal	25.49	66.37	40.43	12.29	2.03	0.07	0.33	0.35
21	Lasbella	25.84	66.52	68.25	11.96	1.68	0.05	0.34	0.19

The basic assumptions (e.g., homogeneity, independence and stationarity) of the data were checked by using the Mann-Whitney *U* test, Wald-Wolfowitz test, and Spearman rank correlation test, respectively. The finding relevant to fundamental assumptions is given in the results and discussion section. Overall, the tests' results demonstrate that the data meet the basic prerequisites and can be used for further analysis.

### 2.2 Linear moments

Initially [42], developed linear moments (LMs) for sample data and distributions through the expectation of a linear combination of order statistics. From a computational point of view, LMs are considered superior to ordinary moments. The shape of the distributions can also be judged through LMs. LMs also offer the measure of dispersion, location, skewness and kurtosis. In general, they work more efficiently than the other methods when dealing with small sample sizes and outliers [42].

In addition, the LMs in terms of a linear combination of probability-weighted moments (PWMs). Let Y be a random variable with a cumulative distribution function H(.), the PWMs are defined as

$$\Lambda_{r}=E\left[Y{\left\{H\left(Y\right)\right\}}^{r}\right],r=0,1,2,...$$

The LMs are defined as

(1) $$\kappa_{r+1}=\sum_{j=0}^{r}Z_{r,j}\Lambda_{r},r=0,1,2,...,\left(n-1\right)$$

where $Z_{r,j}={\left(-1\right)}^{r-j}\left(\begin{array}{l} r\\ j \end{array}\right)\left(\begin{array}{l} r+j\\ j \end{array}\right)=\frac{{\left(-1\right)}^{r-j}\left(r+j\right)!}{{\left(j!\right)}^{2}\left(r-j\right)!}$. Using expression (1), the first four population moments in terms of PWMS are given as

$$\kappa_{1}=\Lambda_{0}$$

$$\kappa_{2}=2\Lambda_{1}-\Lambda_{0}$$

$$\kappa_{3}=6\Lambda_{2}-6\Lambda_{1}+\Lambda_{0}$$

$$\kappa_{4}=20\Lambda_{3}-30\Lambda_{2}+12\Lambda_{1}-\Lambda_{0}$$

where $\kappa_{1}$ and $\kappa_{2}$ are the measure of location and dispersion, respectively. The linear moments based measures of coefficient of variation, skewness and kurtosis are defined as:

Coefficient of variation: Π=κ2κ1.

Skewness: Π3=κ3κ2 , and kurtosis: Π4=κ4κ2.

In general, we used unbiased estimator $\lambda_{r}$ of $\Lambda_{r}$ of PWMS, that is

(2) $$\lambda_{r}=\frac{1}{n}\sum_{k=r+1}^{n}\frac{\left(k-1\right)\left(k-2\right)...\left(k-r\right)}{\left(n-1\right)\left(n-2\right)...\left(n-r\right)}y_{k:n}$$

Using equation (2), the first four sample LMs with combination of sample PWMs are given in the following forms:

$$l_{1}=\lambda_{0}$$

$$l_{2}=2\lambda_{1}-\lambda_{0}$$

$$l_{3}=6\lambda_{2}-6\lambda_{1}+\lambda_{0}$$

$$l_{4}=20\lambda_{3}-30\lambda_{2}+12\lambda_{1}-\lambda_{0}$$

The sample based LMs ratios are defined as $\pi =l_{2}/l_{1},\pi_{3}=l_{3}/l_{2},\pi_{3}=l_{4}/l_{2}$, where $\pi ,\pi_{3}$ and $\pi_{3}$ are sample based linear measure of coefficient, skewness and kurtosis, respectively.

### 2.3. Linear moments-based regional frequency analysis

To work with RFA, we need to follow the footsteps of Hosking and Wallis [27], the four steps are required for LMs based RFA. The following four steps based on LMs are required:

Step 1 Data cleaning and screening

Step 2 Construction of homogenous regions on the basis of station characteristics or by using statistical tests.

Step 3 Selection of best-fit probability distribution.

Step 4 Parameters estimation of the best-fitted models and accuracy of estimated quantiles.

On the other hand, the ASFA is also used in this study, step 2 is not required when we are dealing with ASFA.

### 2.4 Data cleaning and screening

Data screening is used as a tool to remove the inconsistent station from the study. The LMs-based discordancy measures play a crucial role in the data cleaning and screening. The discordancy measure called $\left(\Phi_{i}\right)$ is defined as

(3) $$\Phi_{i}=\frac{1}{3}{\left(\nu_{i}-\bar{\nu }\right)}^{T}Q^{-1}\left(\nu_{i}-\bar{\nu }\right)\text{,}$$

where $\nu_{i}={\left[\pi^{\left(i\right)}\;\pi_{3}^{\left(i\right)}\;\pi_{4}^{\left(i\right)}\right]}^{T}$ is a vector of LMs ratios, and Q is variance covariance matrix. For a decision point of view, we shall remove the station from the analysis if the resultant value of $\left(\Phi_{i}\right)$ defined in expression (3) exceeds the critical value. In this study, the critical value of $\left(\Phi_{i}\right)$ is set as 3 for all stations. Screening of data is an early try to form homogenous regions.

### 2.5 Formation of homogenous regions

The formation of homogeneous regions is the most crucial step in RFA. When all the stations in a region share some common characteristics such as sewerage zone, mean yearly rainfall, speed of the wind, latitude, longitude, drainage area, and time of occurrences of most significant flood in the year etc., then the region is said to be homogeneous. Many studies have been conducted on different grouping methods, such as geographical convenience, subjective partitioning, objective partitioning, and cluster analysis. In this study, the regions have been constructed by using cluster analysis and heterogeneity tests. Cluster analysis is a multivariate statistical approach used to construct the homogenous. It works by organizing stations into groups, or clusters, on the basis of how closely related they are. Here, we use Ward's method to perform the cluster analysis, interested readers can see for example, [43]. For a higher number of stations in each group/cluster and a smaller range of drainage area, the cluster analysis procedure is considered more appropriate as affirmed by Ref. [43]. Second possibility is to compute the heterogeneity measure ★ of the region which can be constructed on the basis of cluster analysis. Heterogeneity measure ★ is defined as

(4) $$H=\frac{\eta -\mu_{\eta }}{\sigma_{\eta }}\text{,}$$

Where $\eta ={\left[\sum_{i=1}^{N}n_{i}{\left(\pi^{i}-\pi^{R}\right)}^{2}/\sum_{i=1}^{N}n_{i}\right]}^{\frac{1}{2}}$ and $\pi^{R}=\sum_{i=1}^{N}n_{i}\pi^{i}/\sum_{i=1}^{N}n_{i}$ is the weighted standard deviation and regional mean of linear moment based sample coefficient of variation. To determine the heterogeneity, the relative dispersion of linear moment-based CVs of each station is measured using weighted standard deviation as shown in the above expression. In addition, a simulation experiment with four-parameter kappa distribution is performed to the heterogeneity of proposed regions. In detail, the kappa distribution is fitted to sample LMs ratios, and the distribution parameters are estimated analytically. Then, the sample of size 500 is a simulation from the fitted distribution (i.e. Kappa distribution) for each region. The experiment is repeated $10^{2}$ times. Mean and variance is calculated corresponding to every station and is denoted by $\mu_{\eta_{j}}$ and $\sigma_{\eta_{j}}$. We will further rely on equation (4) to differentiate region characteristics. Hence, ★< 1 refers to an adequately homogeneous region, if 1≤H<2, the region is possibly a homogeneous and if ★ >2 the region will become heterogeneous.

### 2.6 Selection of an appropriate distribution

An appropriate model selection is required for constructed homogenous regions. Hosking and Wallis [27] argue that best-fit distribution quantile estimates are more accurate and reasonable. Generally, the distribution with the higher number of parameters comprises less bias and provide a suitable estimation of extreme quantiles. Therefore, the goodness of fit measure is defined as:

$$Z^{dist}=\frac{\pi_{4}^{dist}-\pi_{4}^{R}+B_{4}}{\sigma_{4}}\text{,}$$

where $\pi_{4}^{dist}$ and $\pi_{4}^{R}$ are fitted distribution L-kurtosis and: regional weighted average L-kurtosis, respectively. $B_{4}$ and $\sigma_{4}$ are the bias and standard deviation of $\pi_{4}^{R}$. The smaller value of $Z^{dist}$ is the sign of good fit, which indicates that the true distribution is same as the distribution we are fitting to the data. If the value of $Z^{dist}$ is about close to zero or if $\left|Z^{dist}\right|\leq 1.645$ that is, the fit is recognized to be appropriate.

### 2.7 Inference and robustness of estimated quantiles

This section deals with parameters estimation of the selected appropriate model and evaluation of its accuracy in producing efficient quantiles estimates for all sites in the homogeneous region. Hosking and Wallis, 1997 [27] suggested that the regional LMs algorithm be more practical despite the non-fulfilment of some basic assumptions of the index flood procedure. Simulation based regional quantiles are estimated for several non-exceedance probabilities. Additionally, the quantile estimates of each site could be obtained by scaling Q(.) with an estimate of the scaling factor of $\mu_{i}$ conforming to non-exceedance probability G as follows:

$$\hat{q}\left(G\right)=l_{1}^{\left(i\right)}\hat{Q}\left(G\right)\text{,}$$

where Q(.) is the quantile function of regional frequency distribution, $l_{1}^{\left(i\right)}$ is the sample mean. The designed regional frequency is assessed using two robustness measures, namely relative bias (RB) and relative root mean square error (RRMSE). We implemented the Monte Carlo simulation technique with 10,000 simulations to estimate RB and RRMSE. For more details about assessment measures. The detailed findings are given in the results and discussion section.

## 3 Results and discussion

### 3.1 Basic assumptions

It is necessary to test the fundamental assumptions of the observed data before conducting RFA. On the other hand, the final results could be doubtful without satisfying the basic assumptions of the data. Wald-Wolfowitz, Mann-Whitney, and Spearman's rank correlation tests are used to test the assumptions of independence, homogeneity, and stationarity. For the theoretical background of these procedures, we refer to ([44], chap. 7). In Table 2, the p>0.05 corresponding to each station indicates that the basic assumptions of the data are satisfied and the data of the considered stations can be used for RFA.

**Table 2:** The results of basic assumptions and p-value is given in parenthesis.

Station	location	Wald-Wolfowitz Test	Mann-Whitney test	Spearman Rank Correlation test
1	Chhor	0.72 (0.24)	−0.89 (0.19)	−0.62 (0.27)
2	Hydrabad	0.20 (0.43)	−1.64 (0.05)	−0.61 (0.27)
3	Jiwani	−0.36 (0.36)	−1.43 (0.08)	−1.74 (0.04)
4	Jacobabad	−0.57 (0.28)	−0.15 (0.44)	1.19 (0.12)
5	Karachi.A.P	−0.28 (0.39)	−0.10 (0.46)	0.80 (0.21)
6	Nawabshah	0.28 (0.39)	−1.64 (0.05)	0.09 (0.46)
7	Punjgur	−0.50 (0.31)	−1.63 (0.05)	−1.71 (0.10)
8	Pasni	−1.47 (0.11)	−1.47 (0.07)	1.55 (0.06)
9	Badin	0.46 (0.32)	−1.55 (0.06)	−1.59 (0.06)
10	Gwadar	0.23 (0.41)	−0.08 (0.21)	−0.58 (0.28)
11	Larkana	−0.63 (0.27)	−1.14 (0.13)	−0.43 (0.34)
12	Padidan	0.84 (0.20)	−1.22 (0.11)	0.19 (0.44)
13	Rohri	1.23 (0.11)	−0.52 (0.30)	0.05 (0.48)
14	Ormara	−0.44 (0.33)	−0.60 (0.27)	−0.85 (0.20)
15	Turbat	0.49 (0.31)	−1.40 (0.08)	1.90 (0.03)
16	Karachi	−0.99 (0.16)	−0.81 (0.21)	−1.13 (0.13)
17	Dadu	−0.53 (0.30)	−1.56 (0.06)	−0.55 (0.29)
18	Mirpurkhas	−0.66 (0.26)	−0.97 (0.17)	−0.26 (0.40)
19	Thatta	−0.18 (0.43)	−1.56 (0.06)	−1.57 (0.06)
20	Uthal	−0.43 (0.33)	−2.26 (0.06)	−1.95 (0.07)
21	Lasbella	−0.64 (0.24)	−1.42 (0.08)	−1.04 (0.09)

**Fig. 2:**
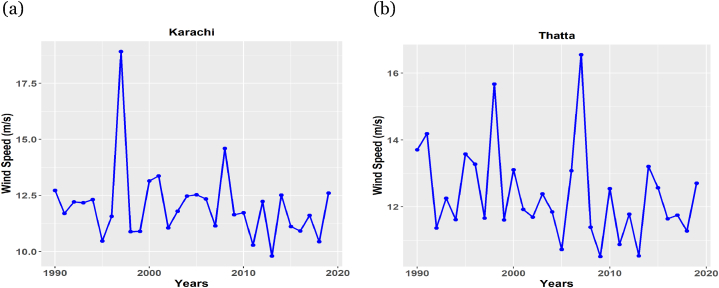
Historigrams of: (a) Karachi station and, (b) Thatta station.

Further to explore the configuration of the DAMWS, time series plots are also constructed. The pattern of DAMWS observation from 1990 to 2019 is the confirmation of accepting the null hypothesis of stationarity. The time series plots corresponding to Karachi and Thatta stations are depicted in Fig. 2 (a) and Fig. 2 (b), respectively. From the plots, it can be noted that there is no upward and downward pattern in the data. Similarly, the other sites also have similar pattern like these two.

### 3.2 Screening of the data using discordancy measure

In RFA, data screening is considered the essential step for discordant stations if any; for this, the most suitable discordancy measure statistic $\Phi_{i}$ is used. The discordancy statistic $\Phi_{i}$ is estimated for every station by assuming all stations lie in one region. The results are given in Table 3. It can be noted that the calculated values of $\Phi_{i}$ for all sites are less than the critical value (i.e., 3) except site Lasbella which has a computed value of 3.05. Therefore, the Lasbella station is declared as discordant. Similar statistical measures have been used by many researchers (see for instance Ref. [13], and references therein).

**Table 3:** Linear moments and discordancy measure.

Station No.	Name	$n_{i}$	$l_{1}$	π	$\pi_{3}$	$\pi_{4}$	$\Phi_{i}$
1	Chhor	30	10.77	0.06	0.02	0.16	1.02
2	Hyderabad	30	12.39	0.05	−0.02	0.12	0.69
3	Jiwani	30	12.11	0.04	0.11	0.09	0.94
4	Jacobabad	30	10.76	0.05	0.15	0.23	0.23
5	Karachi.A.P	30	12.18	0.05	0.17	0.22	0.15
6	Nawabshah	30	11.83	0.05	0.04	0.15	0.26
7	Punjgur	30	15.87	0.05	0.24	0.28	0.95
8	Pasni	30	13.78	0.05	0.19	0.19	0.39
9	Badin	30	12.79	0.05	0.06	0.04	1.86
10	Gwadar	30	12.03	0.04	0.24	0.19	1.86
11	Larkana	30	11.37	0.05	−0.01	0.12	0.58
12	Padidan	30	11.54	0.05	0.07	0.14	0.18
13	Rohri	30	11.75	0.05	−0.12	0.09	1.87
14	Ormara	30	13.52	0.05	0.33	0.25	0.98
15	Turbat	30	16.48	0.04	0.17	0.14	0.61
16	Karachi	30	12.04	0.07	0.25	0.32	1.24
17	Dadu	30	12.07	0.06	0.10	0.18	0.32
18	Mirpurkhas	30	12.29	0.05	0.08	0.23	1.50
19	Thatta	30	12.37	0.06	0.24	0.20	0.65
20	Uthal	30	12.29	0.07	0.33	0.35	2.04
21	Lasbella	30	11.96	0.08	0.30	0.22	3.05*

In addition, some other quantities are also reported in Table 3. For instance, n is the number of observed data points in the respective station. Further, $l_{1}$, $\pi ,\pi_{3}$ and $\;\pi_{4}$ indicate the first sample moment (i.e., sample mean), L-CV, linear skewness and linear kurtosis, respectively. Turbat station has the highest mean with a relatively low L-CV in the data. Most stations have positively skewed data, while three stations, namely Hyderabad, Larkana and Rohri have negatively skewed data.

### 3.3 Regions formation

After removing the discordant station from the data, the next step is to construct the homogeneous regions. The term “homogeneous region” implies that the sites alliance into homogenous regions by their mutual characteristics, such as geographical, hydrological, or others. In this study, we use heterogeneity measure ★ and cluster analysis for developing the regions. By following the procedure given in (subsection “formation of homogeneous regions”), $H_{1},H_{2}$ and $H_{3}$ heterogeneity measures based on L-Cv, L-Skewness and L-Kurtosis are calculated by keeping all stations in one region. Table 4 displays the results of heterogeneity measures. The statistic value of $H_{1}$ <1 and $H_{3}$ <1 represent that all station form exact homogenous region. Further, the statistic value of $H_{2}$ <2 also indicates that all the stations might be existing in one homogenous region. The statistic $H_{2}$ >1 also suggests to construct more than one homogenous region. For further clarification about the formation of homogenous regions, we perform the hierarchical cluster analysis.

**Table 4:** Heterogeneity statistic for homogeneous regions.

Heterogeneity measure $H_{1}$	0.53
Heterogeneity measure $\;H_{2}$	1.08
Heterogeneity measure $H_{3}$	0.47

**Fig. 3:**
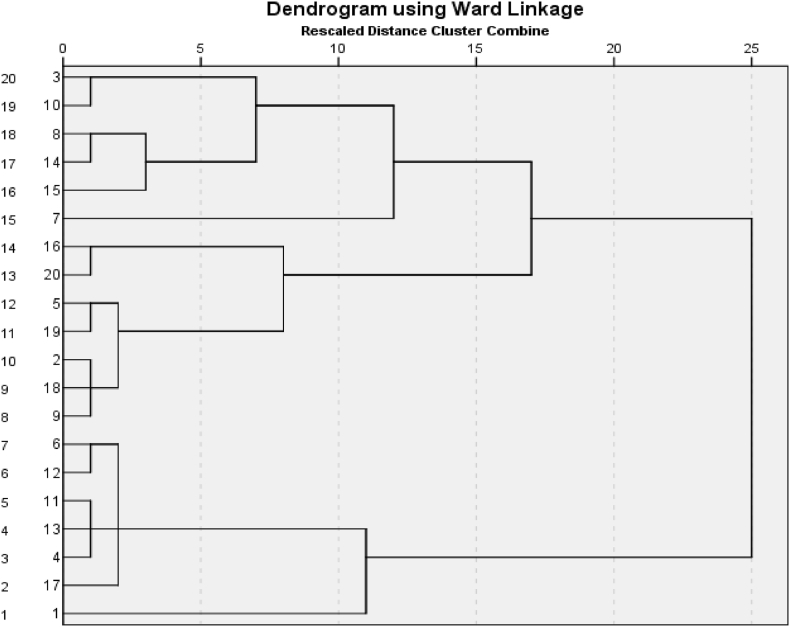
Dendrogram for cluster I and II.

Hierarchical clustering is considered a very efficient tool for developing homogenous regions. Fig. 3 displays the dendrogram constructed through hierarchical cluster analysis (Ward's method) using the information from all 20 stations. Only two clusters are identified in Fig. 3, cluster one (called Region I) has 13 stations and cluster two (called Region II) has 7 stations. The LMs-based results of the stations inside both regions are also provided in Table 3. Further, Region I shows a relatively higher slope. To verify the homogeneity measure inside the cluster and/or the regions, again LMs ratios and discordancy measures $\Phi_{i}$ are calculated separately for each station in both regions. Table 5 reports the results of stations involved in both regions. The results clearly show that no station is discordant in both regions. This means that these stations can be used for RFA and quantile estimation. Furthermore, the heterogeneity measures $H_{1},H_{2}$ and $H_{3}$ are again estimated for both regions individually. Results are shown in Table 5. Table 6 validates our constructed regions and shows that both regions are homogenous. After developing the homogenous, the next step is to decide the best-fitted model for each region. The details regarding the goodness of fit of the probability model are given in a subsequent section.

**Table 5:** LMs and Discordancy Measure corresponding to Region I and Region-II.

Station No.	Name	$n_{i}$	$l_{1}$	π	$\pi_{3}$	$\pi_{4}$	$\Phi_{i}$
Region I							
9	Badin	30	12.78	0.05	0.05	0.04	1.82
18	Mirpurkhas	30	12.29	0.05	0.08	0.23	1.69
2	Hydrabad	30	12.39	0.05	−0.02	0.12	1.73
19	Thatta	30	12.37	0.06	0.24	0.20	0.97
5	KarachiA.P	30	12.18	0.05	0.17	0.22	0.07
20	Uthal	30	12.29	0.07	0.33	0.35	1.69
16	Karachi	30	12.04	0.07	0.25	0.32	0.89
7	Punjgur	30	15.87	0.05	0.24	0.28	0.52
15	Turbat	30	16.48	0.04	0.17	0.14	0.34
14	Ormara	30	13.52	0.05	0.33	0.25	0.92
8	Pasni	30	13.78	0.05	0.19	0.19	0.18
10	Gwadar	30	12.03	0.04	0.24	0.19	1.6
3	Jiwani	30	12.11	0.04	0.11	0.09	0.59
Region II							
6	Nawabshah	30	11.83	0.05	0.04	0.15	0.06
12	Padidan	30	11.54	0.05	0.07	0.14	1.14
11	Larkana	30	11.37	0.05	−0.01	0.12	0.48
13	Rohri	30	11.75	0.05	−0.11	0.09	1.36
4	Jacobabad	30	10.76	0.05	0.15	0.23	1.86
17	Dadu	30	12.07	0.06	0.10	0.18	1.13
1	Chhor	30	10.77	0.06	0.02	0.16	0.97

**Table 6:** Heterogeneity statistic for homogeneous Region-I and Region-II.

Heterogeneity measure	Region I	Region II
$H_{1}$	0.31	−0.78
$H_{2}$	−0.22	−0.48
$H_{3}$	−0.18	−1.23

The ZDIST statistic suggested by Hosking and Wallis [27] are used to select the best fitted distribution among five distributions namely Generalized Logistic (GLO), Generalized Pearson type III (PE3), Generalized Extreme Value (GEV), Generalized Normal (GNO), and Generalized Pareto (GPA). According to Ref. [27], If the critical value i.e., |Z^Dist^| ≤ |Z_0.05_| = 1.64, we can reject the hypothesis of homogeneity. The results linked with Z^Dist^ statistic are given in Table 7. Hence, the Z^Dist^ statistic indicate that the GLO distribution is an appropriate for modelling wind data in both regions. Furthermore, the second best distribution to region one is GEV and to region two is GNO. For more clarification, we perceive fitting through LMs ratio diagram.

**Fig. 4:**
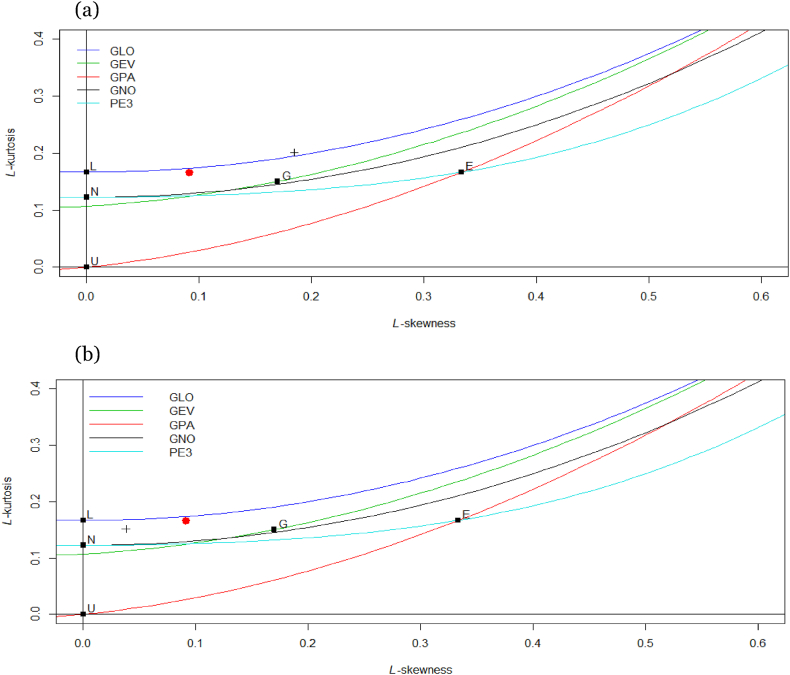
(a) L-moment ratio diagram for Region I (b) L-moment ratio diagram for Region II.

LMs ratio diagram is a graphical tool which is very popular to select best model in extreme value theory. In the LMs ratio diagram (see Fig. 4 (a, b)), the red dot sign represents the sample regional skewness and kurtosis ratio as shown on the curve of the distributions. The red dot is existing very close to GLO distribution curve in both regions. This clearly indicate that the GLO distribution is more appropriate for DAMWS in both regions.

### 3.4 Parameter estimates and regional quantiles

This section deals with the estimation of regional parameters of the fitted distribution. Also, the regional quantiles corresponding to different return periods *T* are estimated by using regional parameter estimates of the distribution in the quantile function of the distribution. Further, the return periods can be written in terms of exceedances probability (i.e., p = 1/T). On the other hand, the non-exceedances probability regarding period T is p = 1-1/T. The results related to parameter estimates and return levels for both regions are shown in Table 8.

**Table 7:** Best fitted distribution for homogenous Region I and Region II. Absolute values are given in parenthesis.

Region	Z^GLO^ | Z^GLO^|	Z^GEV^ |Z^GEV^|	Z^GNO^ | Z^GNO^|	Z^PE3^ | Z^PE3^|	Z^GPA^ | Z^GPA^|
I	−0.85 (0.85^a^)	−2.43 (2.43)	−2.71 (2.71)	−3.35 (3.35)	−6.02 (6.02)
II	−0.62) (0.62)	−1.49 (1.49)	−1.07 (1.07)	−1.09 (1.09)	−5.45 (5.45)

^a^Indicate the best fit distribution.

**Table 8:** Regional based parameters and quantile of best-fitted distribution.

Region	Parameters regional quantiles estimates with non-exceedance probability G											
	Dist.	ε	α	k	0.50	0.80	0.90	0.95	0.98	0.99	0.998	0.999
					2	5	10	20	50	100	500	1000
Ι	GLO	0.98	0.05	−0.18	0.98	1.06	1.11	1.17	1.26	1.33	1.54	1.65
ΙΙ	GLO	0.99	0.05	−0.04	0.10	1.07	1.11	1.15	1.21	1.25	1.35	1.40

**Fig. 5:**
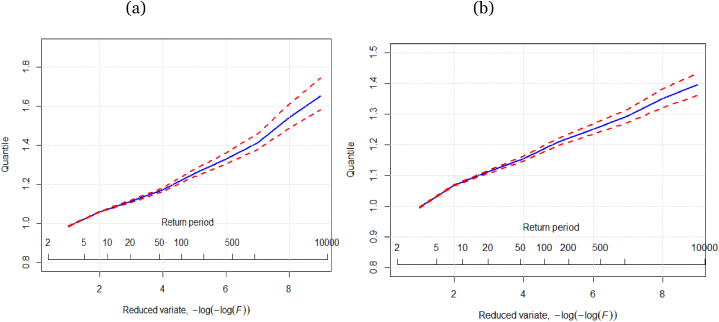
(a) GLO distribution regional growth curve for Region I (b) GLO distribution regional growth curve for Region II.

Additionally, the regional growth curve with error bounds for GLO distribution has been produced for both regions. The regional growth curve for any distribution is pretentious by the magnitude of L-CV and L-skewness. It is shown in Fig. 5 (a, b) that GLO distributions have a good slope. It is portrayed in Fig. 5 that the growth curves with error bounds of GLO for both regions have similar behaviour up to 20 years return periods. Errors bounds for the GLO growth curve in both regions at a higher level are relatively narrow.

**Fig. 6:**
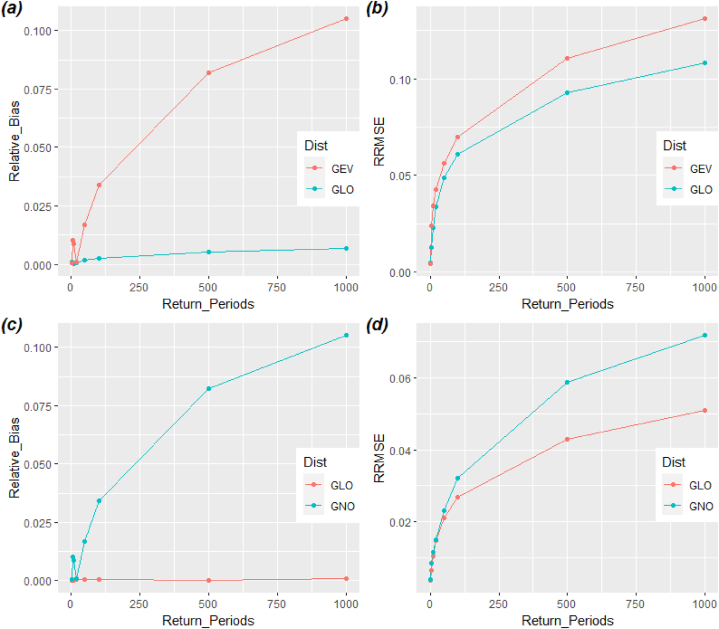
Graphical display of assessment measures for best-fit distributions. (a, b) Relative bias and relative root mean square error for GLO and GEV distribution corresponding to region I, (c, d) Relative bias and relative root mean square error for GLO and GNO distribution corresponding to region II.

To choose the most reliable distribution among the distributions standing at first and second position in Table 7. We practiced a Monte Carlo simulation algorithm proposed by Meshgi and Khalili (2009) for design flood estimates to assess the efficiency of RFA distribution through relative bias (RB) and relative root mean square error (RRMSE). The simulation results of RB and RRMSE corresponding to different return periods are shown Fig. 6. Fig. 6 (a, b) is representing the region I and it show that GLO distribution have smaller RB and RRMSE for the all return periods than GEV distribution. On the other hand, Fig. 6 (c, d) represent RB and RRMSE related to region II. Similar to region I, the GLO distribution again having the lowest RB and RRSME than GNO distribution. Overall, Fig. 6 specify that GLO distribution is outperforming in both regions than GNO and GEV distribution.

### 3.5 At-site quantiles estimation

It is not possible in practice to use regional quantiles until we calculate at-site quantiles. For each station, at-site quantiles can be derived by multiplying the regional quantiles by the sample mean or median of that particular station, i.e., $\hat{q}\left(G\right)=l_{1}^{\left(i\right)}{\hat{Q}}_{i}\left(G\right)$, where G = 1/T.

In this regional study, estimates of at-site quantiles are produced for both regions using the quantile function of best-fitted distribution to regions (i.e., GLO) while sample means and medians play a role as scale factors. The result of the estimated quantile for Region I and Region II are presented in Table 9 and Table 10. From the findings given in Table 9, Table 10, we notice that the quantiles estimate corresponding to a region I are slightly higher for some stations, namely Mirpurkhas, Thatta and Karachi airport when using the median as a scaling factor. Similarly, the quantile estimates related to the Rohri station are slightly higher for smaller return periods when the median plays a scaling factor. This may happen due to a higher median than the mean. On the other hand, all the stations of both regions have higher quantiles when using mean as scale factor except Mirpurkhas, Thatta, Karachi airport and Rohri (see, Table 9, Table 10).

**Table 9:** At site Quantile Estimate corresponding 2-, 5-, 10-, 20-, 50-, 100-, 500-, and 1000- years return periods at Region I using mean and [median] as scaling factors.

Badin	GLO	12.59 [12.44]	13.56 [13.38]	14.25 [14.07]	14.98 [14.79]	16.07 [15.87]	17.01 [16.80]	19.70 [19.45]	21.12 [20.86]
Mirpurkhas	GLO	12.10 [12.16]	13.03 [13.09]	13.69 [13.76]	14.39 [14.47]	15.45 [15.52]	16.35 [14.43]	18.93 [19.02]	20.30 [20.39]
Hydrabad	GLO	12.20 [12.16]	13.14 [13.10]	13.81 [13.76]	14.52 [14.47]	15.57 [15.52]	16.49 [16.43]	19.09 [19.03]	20.47 [20.40]
Thatta	GLO	12.18 [12.71]	13.12 [12.60]	13.78 [13.24]	14.49 [13.93]	15.55 [14.94]	16.46 [15.81]	19.06 [18.31]	20.43 [19.63]
KarachiA.P	GLO	12.00 [11.93]	12.92 [12.85]	13.58 [13.50]	14.27 [14.20]	15.31 [15.23]	16.21 [16.12]	18.77 [18.66]	20.12 [20.01]
Uthal	GLO	12.10 [11.91]	13.03 [12.83]	13.70 [13.48]	14.40 [14.17]	15.45 [15.20]	16.35 [16.09]	18.93 [18.63]	20.30 [19.98]
Karachi	GLO	11.86 [11.58]	12.76 [12.47]	13.41 [13.11]	14.11 [14.78]	15.13 [14.78]	16.02 [16.65]	18.54 [18.12]	19.88 [19.43]
Punjgur	GLO	15.63 [15.22]	16.83 [16.39]	17.68 [17.22]	18.59 [18.11]	19.94 [19.43]	21.11 [20.56]	24.45 [23.81]	26.21 [25.53]
Turbat	GLO	16.24 [16.16]	17.48 [17.40]	18.37 [18.28]	19.32 [19.22]	20.72 [20.62]	21.93 [21.83]	25.40 [25.27]	27.23 [27.10]
Ormara	GLO	13.32 [13.08]	14.34 [14.08]	15.07 [14.79]	15.84 [15.56]	16.99 [16.69]	17.97 [17.66]	20.83 [20.45]	22.33 [21.93]
Pasni	GLO	13.57 [13.52]	14.61 [14.56]	15.35 [15.30]	16.14 [16.09]	17.32 [17.26]	18.33 [18.27]	21.23 [21.15]	22.76 [22.68]
Gwadar	GLO	11.85 [11.58]	12.76 [12.47]	13.40 [13.11]	14.09 [13.78]	15.12 [14.78]	16.00 [15.65]	18.53 [18.12]	19.87 [19.43]
Jiwani	GLO	11.93 [11.82]	12.85 [12.73]	13.50 [13.72]	14.19 [14.06]	15.23 [15.08]	16.12 [15.97]	18.66 [18.49]	20.01 [19.82]

**Table 10:** At site quantile estimates corresponding 2-, 5-, 10-, 20-, 50-, 100-, 500-, and 1000- years return periods at Region II using mean and [median] as scaling factors.

Site Name	Dist.	2	5	10	20	50	100	500	1000
Nawabshah	GLO	11.79 [11.79]	12.65 [12.65]	13.17 [13.16]	13.66 [13.66]	14.30 [14.30]	14.80 [14.80]	15.99 [15.99]	16.52 [16.52]
Padidan	GLO	11.51 [11.29]	12.34 [12.11]	12.84 [12.60]	13.33 [13.07]	13.95 [13.69]	14.44 [14.17]	15.60 [15.30]	16.12 [16.81]
Larkana	GLO	11.34 [11.27]	12.16 [12.09]	12.66 [12.59]	13.13 [13.06]	13.75 [13.67]	14.23 [14.15]	15.37 [15.28]	15.88 [16.79]
Rohri	GLO	11.71 [11.69]	12.56 [12.53]	13.07 [13.05]	13.56 [13.54]	14.20 [14.18]	14.69 [14.67]	15.87 [15.84]	16.40 [14.37]
Jacobabad	GLO	10.72 [10.52]	11.41 [11.28]	11.97 [11.74]	12.42 [12.18]	13.00 [12.76]	13.45 [13.20]	14.53 [14.26]	15.02 [16.73]
Dadu	GLO	12.03 [12.02]	12.90 [12.89]	13.43 [13.41]	13.93 [13.92]	14.59 [14.57]	15.09 [15.08]	16.31 [16.29]	16.85 [16.83]
Chhor	GLO	10.73 [10.65]	11.51 [11.42]	11.98 [11.89]	12.43 [12.34]	13.02 [12.92]	13.47 [13.37]	14.55 [14.44]	15.03 [14.92]

In addition, the standard errors of at-site quantiles estimate for both regions are calculated by using the following equation.

(8) $$Var\left(\hat{Q}\left(G\right)\approx {\left\{x\left(G;\theta_{0}\right)\right\}}^{2}Var\left({\hat{\mu }}_{i}\right)\right)+\mu_{i}^{2}Var\left\{x\left(Q;\theta_{0}\right)\right\}$$

In practice, the term $x\left(G;\theta_{0}\right)$ is substituted by estimates of regional quantiles $\mu_{i}$, with a sample scaling factor, which might be sample mean or sample median [13] The results of standard errors for station of both regions are shown in Table 11 and Table 12 using mean and median as scaling factors, respectively. To determine which scaling factor is relatively superior, we can compare Table 11, Table 12 together. Overall, the standard errors for region I and region II based on mean scaling factor are relatively smaller than the standard errors based on median scaling factor.

**Table 11:** Standard errors of at-site quantile estimates of region I using mean and [median] as scaling factors.

Site Name	Dist.	$Q_{2}$	$Q_{5}$	$Q_{10}$	$Q_{20}$	$Q_{50}$	${\;Q}_{100}$	$Q_{500}$	$Q_{1000}$
Badin	GLO	0.91 [0.91]	1.46 [1.45]	1.96 [1.94]	2.36 [2.34]	2.83 [2.81]	3.17 [3.13]	3.91 [3.87]	4.22 [4.17]
Mirpurkhas	GLO	0.88 [0.90]	1.40 [1.42]	1.89 [1.90]	2.27 [2.29]	2.73 [2.74]	3.05 [3.07]	3.76 [3.78]	4.06 [4.08]
Hyderabad	GLO	0.88 [0.89]	1.41 [1.41]	1.90 [1.90]	2.29 [2.29]	2.75 [2.74]	3.07 [3.07]	3.79 [3.78]	4.09 [4.08]
Thatta	GLO	0.90 [0.96]	1.42 [1.39]	1.91 [1.85]	2.30 [2.22]	2.75 [2.66]	3.07 [3.97]	3.79 [3.66]	4.09 [3.95]
KarachiA.P	GLO	0.88 [0.89]	1.39 [1.40]	1.87 [1.87]	2.26 [2.25]	2.71 [2.70]	3.02 [3.01]	3.73 [3.72]	4.03 [4.01]
Uthal	GLO	0.93 [0.96]	1.44 [1.45]	1.92 [1.92]	2.30 [2.29]	2.76 [2.74]	3.08 [3.05]	3.79 [3.76]	4.09 [4.06]
Karachi	GLO	0.89 [0.91]	1.39 [1.39]	1.87 [1.84]	2.24 [2.21]	2.69 [2.64]	3.00 [2.95]	3.70 [3.63]	3.99 [3.92]
Punjgur	GLO	1.15 [1.14]	1.82 [1.79]	2.44 [2.39]	2.94 [2.88]	3.53 [3.45]	3.94 [3.85]	4.86 [4.75]	5.25 [5.13]
Turbat	GLO	1.18 [1.18]	1.88 [1.88]	2.53 [2.52]	3.05 [3.04]	3.66 [3.65]	4.08 [4.07]	5.04 [5.02]	5.44 [5.42]
Ormara	GLO	0.98 [0.99]	1.55 [1.54]	2.08 [2.06]	2.51 [2.48]	3.01 [2.96]	3.36 [3.31]	4.14 [4.08	4.47 [4.41]
Pasni	GLO	0.96 [1.00]	1.57 [1.57]	2.11 [2.11]	2.55 [2.55]	3.06 [3.05]	3.42 [3.41]	4.21] [4.21]	4.55 [4.54]
Gwadar	GLO	0.86 [0.85]	1.37 [1.35]	1.84 [1.81]	2.22 [2.18]	2.67 [2.61]	2.98 [2.92]	3.67 [3.60]	3.97 [3.89]
Jiwani	GLO	0.86 [0.86]	1.38 [1.37]	1.85 [1.84]	2.24 [2.22]	2.68 [2.66]	3.00 [2.98]	3.70 [3.67]	4.00 [3.96]

**Table 12:** Standard errors of at-site quantile estimates of region II using mean and [median] as scaling factor.

Site Name	Dist	$Q_{2}$	$Q_{5}$	$Q_{10}$	$Q_{20}$	$Q_{50}$	${\;Q}_{100}$	$Q_{500}$	$Q_{1000}$
Nawabshah	GLO	0.73 [0.74]	0.98 [0.99]	1.22 [1.23]	1.44 [1.45]	1.74 [1.74]	1.95 [1.96]	2.46 [2.47]	2.69 [2.69]
Padidan	GLO	0.71 [0.71]	0.95 [0.94]	1.19 [1.17]	1.41 [1.39]	1.69 [1.67]	1.90 [1.88]	2.40 [2.37]	2.62 [2.58]
Larkana	GLO	0.70 [0.71]	0.94 [0.94]	1.17 [1.17]	1.39 [1.39]	1.67 [1.66]	1.88 [1.88]	2.37 [2.36]	2.58 [2.57]
Rohri	GLO	0.72 [0.74]	0.97 [0.98]	1.21 [1.22]	1.43 [1.44]	1.72 [1.73]	1.94 [1.94]	2.45 [2.45]	2.67 [2.67]
Jacobabad	GLO	0.67 [0.67]	0.89 [0.89]	1.11 [1.10]	1.32 [1.30]	1.58 [1.56]	1.78 [1.76]	2.24 [2.21]	2.45 [2.41]
Dadu	GLO	0.76 [0.77]	1.00 [1.02]	1.25 [1.26]	1.48 [1.49]	1.78 [1.79]	2.00 [2.01]	2.52 [2.53]	2.75 [2.75]
Chhor	GLO	0.67 [0.67]	0.90 [0.91]	1.12 [1.12]	132 [132]	1.59 [1.58]	1.78 [1.78]	2.25 [2.24]	2.45 [2.44]

## 4 Conclusions

This study investigates the RFA of DAMWS observed at twenty-one sites over the Baluchistan and Sindh provinces of Pakistan. The results confirm that the wind records fulfil the underlying assumptions of independence, homogeneity, and stationarity. The discordancy measure recommends that no other station is discordant except Lasbella station. Therefore, except Lasbella station, all other stations can be considered further for RFA. Initially, the twenty stations are considered homogeneous based on their geographic location. The regional heterogeneity test further demonstrates its homogeneity, which shows that the nine stations constitute a single homogenous region. In addition, we further run a cluster analysis which suggests that all twenty stations formed two homogenous regions. The best-fit distribution was decided for both regions via ZDIST statistic criteria and L-moments ratio diagram. We observe that the GLO distribution out of the several probability distributions was best fitted to both homogeneous regions. The robustness of GLO distribution as compared with GEV distribution is also assessed by using RB and RRMSE assessment measures. The results imply that RB and RRMSE are smaller for GLO distribution. Hence, GLO distribution is considered an excellent choice for regional analysis of wind records.

In the second step, we calculated the at-site quantiles by multiplying the regional quantiles with sample means and medians as scaling factors. For at-site quantiles, the performance of GLO distribution is quite reasonable with both scaling factors. In addition, we calculated the standard errors of these at-site quantiles. The findings can be compared with the quantiles and respective standard errors calculated by through at-site frequency analysis for both regions. We notice that the quantiles estimate corresponding to a region I are slightly higher for some stations, namely Mirpurkhas, Thatta and Karachi airport when using the median as a scaling factor. Similarly, the quantile estimates related to the Rohri station are slightly higher for smaller return periods when the median plays a role as a scaling factor in region II. Overall, the standard errors for region I and region II based on the mean scaling factor are relatively smaller than the standard errors based on the median scaling factor. In order to diminish losses because of heavy wind speeds, the estimated quantiles of DAMWS through the quantile function of GLO distribution can be used for policy implications in codifying the wind load for different standardized structural designs. Also, the finding of this study could be helpful for engineers in the installation of wind turbines in these considered areas or at ungagged sites. This study can further be enhanced by considering the Bayesian and spatial phenomena in the regionalization process.

## Data availability statement

The data used in this study is available freely and can be downloaded from the GitHub repository named “RM_Underfloodindexprocedure” by opening the given link. https://github.com/touqeerahmadunipd/. In addition, the complete source code used for this study can be acquired from the corresponding author on reasonable request.

## CRediT authorship contribution statement

**Ishfaq Ahmad:** Validation, Supervision, Investigation, Conceptualization. **Touqeer Ahmad:** Writing – review & editing, Writing – original draft, Software, Methodology, Formal analysis, Data curation. **Usman Shahzad:** Writing – review & editing, Writing – original draft, Formal analysis. **Muhammad Athar Ameer:** Writing – original draft, Validation, Methodology, Data curation. **Walid Emam:** Writing – review & editing, Writing – original draft, Funding acquisition, Formal analysis. **Yusra Tashkandy:** Writing – original draft, Validation, Methodology, Funding acquisition. **Zanib Badar:** Writing – review & editing, Writing – original draft, Methodology.

## Declaration of competing interest

The authors declare that they have no known competing financial interests or personal relationships that could have appeared to influence the work reported in this paper.
